# Income Adequacy, Pre-Sleep Arousal, and Multidimensional Sleep Health Among Older Adults

**DOI:** 10.1093/geroni/igaf122.3099

**Published:** 2025-12-31

**Authors:** Caitlan Tighe, Brooke Sullivan, Grace Lynch, Declan Miles

**Affiliations:** Providence College, Providence, Rhode Island, United States; Providence College, Providence, Rhode Island, United States; Providence College, Providence, Rhode Island, United States; Providence College, Providence, Rhode Island, United States

## Abstract

Existing literature indicates income adequacy is associated with general health perceptions among older adults; less is known about its association with sleep health. We investigated if older adults’ multidimensional sleep health differs by income adequacy and to evaluate if income adequacy relates to sleep health indirectly through experiences of pre-sleep arousal. We analyzed data from a cross-sectional online survey study of participants recruited from Prolific. Income adequacy was assessed with a self-report item wherein respondents indicated the extent of difficulty they have paying for basic necessities. The Pre-Sleep Arousal Scale assessed cognitive and somatic pre-sleep arousal. The RU-SATED v4.0 questionnaire assessed multidimensional sleep health. Aims were evaluated with an independent samples t-test and regression-based parallel mediation analysis. The sample included 179 adults aged 65-82 years (M = 69.66, SD = 4.20). On average, individuals who reported difficulty paying for basic necessities had worse multidimensional sleep health (M = 12.95, SD = 4.13) than those who did not endorse difficulty (M = 14.89, SD = 3.96), t(177)=3.10, p=.001. Income adequacy was associated with multidimensional sleep health indirectly, B=-.94, 95% CI [-1.62, -.39], through cognitive arousal, B=-.87, SE=.32, 95% CI [-1.59, -.31], but not somatic arousal, B=-.07, 95% CI [-.46, .24]. These findings suggest that, in older adults, feeling unable to pay for basic necessities is associated with more pre-sleep cognitive arousal which is, in turn, associated with worse multidimensional sleep health. Prospective research is needed to clarify this association and determine if sleep health interventions that are cognitively-oriented and consider social determinants of health are beneficial for older adults experiencing income inadequacy.

